# Mental Healthcare Utilization among Homeless People in the Greater Paris Area

**DOI:** 10.3390/ijerph17218144

**Published:** 2020-11-04

**Authors:** Valérie Dauriac-Le Masson, Alain Mercuel, Marie Jeanne Guedj, Caroline Douay, Pierre Chauvin, Anne Laporte

**Affiliations:** 1 Département d’Information Médicale, GHU Paris Psychiatrie & Neurosciences, 75014 Paris, France; 2 SMES, GHU Paris Psychiatrie & Neurosciences, 75014 Paris, France; a.mercuel@ghu-paris.fr; 3 CPOA, GHU Paris Psychiatrie & Neurosciences, 75014 Paris, France; mj.guedjbourdiau@gmail.com; 4 Observatoire du Samusocial de Paris, 75012 Paris, France; caroline.douay@free.fr; 5 INSERM, Sorbonne Université, Institut Pierre Louis d’épidémiologie et de Santé Publique (IPLESP), Department of Social Epidemiology, 75012 Paris, France; pierre.chauvin@inserm.fr; 6 Santé publique France, French National Public Health Agency, 94410 Saint-Maurice, France; anne.laporte@santepubliquefrance.fr

**Keywords:** healthcare utilization, psychiatry, homeless people

## Abstract

The healthcare utilization of homeless people is generally considered insufficient, and studies often suffer from methodological bias (institutionalized vs. street samples). To adapt public health policies in France, epidemiological data on this population are scarce. The objective of this study was to analyze the use of psychiatric care by homeless people with mental health problems in the Greater Paris area and to define the factors influencing this use. The data were from the SAMENTA survey performed in 2009 with a representative random street sample of 859 homeless people from the Greater Paris area. The survey studied the use of psychiatric care (lifelong use, current follow-up, discontinuation of follow-up and treatment) and factors potentially associated with this use for people with a diagnosis of a psychotic, mood or anxiety disorder, with the diagnosis established with an original survey device. Because of our complex sampling design, we describe data for only a weighted estimated prevalence, weighted estimation of the number of people in the population (N) and unweighted total subgroup studied in the survey (*n*). Among 840 homeless people with useable data, 377 (N = 9762) had a psychiatric disorder. The use of whole-life care for these people may seem high, estimated at 68.7%, but few people were followed up for their disorders (18.2%); individuals with a psychotic disorder were more frequently followed up (36.5%) than others were (*p* < 0.05). Among those followed up (*n* = 86, N = 1760), 63.0% were taking medication. Access to care for these people seemed preserved, but the maintenance of care seemed problematic; indeed, among people with a lifelong whole use of care (*n*= 232, N = 6705), 72.3% could be considered to have discontinued care. The factors that improved lifetime health service utilization or follow-up were socio-demographic (age < 42 years, more educated), social (with social security coverage, not living in a hotel), and medical (psychotic disorder, personality disorder, suicide risk, somatic chronic illness, perception of mental suffering). Improving the care of homeless people with psychiatric disorders requires improving access to care for those isolated from the health system (in particular those living in hotels) and to guarantee continuity of care, by adapting the organization of the care system and promoting social rehabilitation.

## 1. Introduction

In many countries, mental illnesses are often insufficiently treated, and access to care is deemed insufficient [1,2,3]. The reasons given in these papers are cost, availability of services and organization between primary and specialized care [1,2,3,4]. Socio-cultural factors such as knowledge and beliefs about mental illness also play a role in the underutilization of the health system [4,5,6]. In parallel with these organizational and socio-cultural reasons, several factors inherent in the disease or the socio-demographic characteristics of people with mental illness themselves have been found associated with less use of psychiatric care: male sex [2,7,8,9,10], extreme age (young or old) [2,7,8,9], married [7,8,9], low educational level [2,7,9], lack of psychiatric comorbidity [7,11], less severe psychiatric disorders [2,8], low income [9], and lack of perception of the need for care [12].

Estimates of mental illness prevalence among homeless populations vary widely between studies, due to methodological difficulties [13,14], depending on the nature of the sample (institutionalized vs. street sample). However, the prevalence of psychiatric disorders is often described as being higher in homeless people than in the general population [15,16], including addictive disorder [17,18]. However, according to their living conditions, homeless people must satisfy priorities other than their health needs (they are primarily oriented toward survival: finding a refuge for the night, finding food, taking care of hygiene) [19]. Access to healthcare can also be hampered by economic [20,21], social (fear of stigma) [22], or organizational barriers (the schedules of the structures, the complexity of the organization of the care system) [21,23]. A Canadian study estimated that 1 in 6 homeless people in Toronto had unmet healthcare needs [24].

Thus, although the prevalence of psychiatric disorders seems higher for homeless people than the general population, the former also more often forego care [25] and underuse the healthcare system [26,27]. One American study estimated unmet mental health needs at 21% [28].

Few studies have analyzed the use of psychiatric care by homeless people in France [15] and the factors that could be a barrier or a facilitator in their access to care. These are fundamental questions for public health actors in a context in which the most precarious mental health issue is emphasized in health policies [26,29].

To answer these questions and help healthcare decision-makers improve both the dimensioning of the healthcare offerings in relation to needs and access to the healthcare system, we used the SAMENTA survey data [30] to first study the use of psychiatric care by homeless people of the Greater Paris area (France), then factors positively or negatively associated with this access—that is, the factors likely to constitute barriers to care or to conversely facilitate care.

## 2. Materials and Methods

### 2.1. Population and Data Collection

The SAMENTA study was conducted in 2009 in the Greater Paris area to estimate the prevalence of psychiatric disorders among homeless people. The general methodology of the survey is described elsewhere [31]. The study’s population includes French-speaking adults who had slept at least once in a place not intended for human habitation (street, squat, train station, etc.) or who had been taken in by an organization providing free or low-cost housing services within the five days preceding the survey, as well as those encountered in day-centers and those frequenting hot meal distribution points. The study surveyed 859 people, sampled by a three-stage random sampling from different housing services (short- and long-stay centers, hotels, parent–child centers), day-centers and hot meal distribution points, who were interviewed between February and April 2009, constituting a representative sample of homeless people. 

The SAMENTA study involved administration of a detailed questionnaire on personal and family history, past and present social situation, living and housing conditions, health and use of health services at the time of the survey. The clinical diagnoses of psychiatric pathologies were established by use of an original survey device: a pair of specifically trained investigators (a professional lay interviewer and a clinical psychologist) interviewed each person for an average of 1 h. Psychiatrists were then able to make a diagnostic hypothesis from (1) the result of a structured clinical interview (Mini-International Neuropsychiatric Interview (MINI plus v5.0)), which generated diagnoses according to the Diagnostic and Statistical Manual of Mental Disorders, 4th revision; (2) life events from the questionnaire; (3) elements of the open clinical interview conducted by the clinical psychologist. For the present study, we selected the subgroup of people with a psychiatrist’s diagnosis of one of the following according to codes in the International Classification of Diseases, 10th revision: (1) psychotic disorder (F20-F29), (2) severe mood disorder (F30; F31; F33.3; F33.2; F32.3), (3) anxiety disorder (F40-F49) and (4) non-severe mood disorder (F32.0; F32.1; F32.2; F32.8; F32.9; F33.0; F33.1; F33.4; F34; F38). Only 26 individuals presented dual diagnoses. To avoid collinearity in our regression model, for these cases, we retained a principal diagnosis based on statistical and clinical criteria defined by the psychiatrist. Hence, 7 people with a diagnosis of severe mood disorders and anxiety were considered to have “severe mood disorders”, 17 people with non-severe mood disorders and anxiety were considered to have “anxiety disorders” and 2 people with psychotic disorders and severe mood disorders or not were considered to have “psychotic disorders”.

### 2.2. Statistical Analysis

The use of care was studied in four axes:

- Lifetime use of care for mental health reasons (already consulted a health professional or took medication according to a medical prescription for this reason, or hospitalized in a psychiatry unit)
- Current follow-up (answering the question “Are you currently being followed for mental health reasons?”)
- A break with the healthcare system (lack of current follow-up for people who had care in the past)
- Non-compliance with treatment (answering the question “Have you ever stopped mental health treatment before the end of it?”).

For each person surveyed, a sampling weight was calculated by using the inverse of the product of the inclusion probabilities at each stage of the sampling design. This weight was modified to take into account the heterogeneous use of services by using the generalized weight share method [32]. All statistical analyses and estimates took into account the complex sampling design.

We present in our results (text and tables) the weighted estimated prevalence (percentages) of the use of care, weighted estimation of the number of people in the population (N) and unweighted data for the total subgroup studied in the survey (*n*). Owing to our complex sampling design, the unweighted class group corresponding to prevalence is never shown.

To determine the factors associated or not with care use and follow-up variables, we used a bivariate analysis, testing the following factors: (1) socio-demographic factors (age (18−30; 31−41; 42−82 years), sex (female/male), educational level (secondary or primary), “solitary life” (living alone or with other people)); (2) social factors (social security coverage (yes/no), working (yes/no), previously worked (yes/no), place of accommodation (hotel/emergency facility/reintegration), income (yes/no), length of life without personal accommodation (≥ 4 or <4 years), social support (average number of people to turn to)); (3) co-morbidities (personality disorders (yes/no), alcohol/drug addiction (yes/no), cannabis addiction (yes/no), suicidal risk (yes/no), chronic somatic disease (yes/no)] and markers of psychic suffering [declared psychic suffering (yes/no), feeling alone (yes/no), feeling psychic discomfort (yes/no), feeling a functional discomfort of psychic origin (yes/no)). Factors associated with use of psychiatric care with *p* ≤ 0.15 from a bivariate analysis or known from the literature were integrated into a multivariate logistic regression model, estimating odds ratios (ORs) and 95% confidence intervals (CIs). In the final model, we retained only variables whose deletion did not change the likelihood of the model too much. For disruption of care and treatment adherence, the sample size was deemed too small to produce robust statistical models, so data are not shown. Analyses involved using Stata v11 (StataCorp, College Station, TX, USA). *p* < 0.05 was considered statistically significant.

## 3. Results

### 3.1. Lifetime Psychiatric Use of Care

We had complete data for 840 of the 859 homeless people. For 377 homeless people (N = 9762) with a psychotic, mood (severe or not) or anxiety disorder (PMAD), the lifetime psychiatric use of care was estimated at 68.7%. The use of care consisted of 63.6% of consultations with a professional for mental health reasons, 40.1% of mental health disorder medication prescribed by a doctor, 25.2% of psychiatric hospitalizations and 17.7% of compulsory hospitalizations (Table 1). The diagnostic groups did not differ in frequency of consultations or drug consumption. In contrast, for hospitalizations, including compulsory hospitalizations, individuals with psychotic or mild mood disorders were more often hospitalized than were others (*p* < 0.05). Despite these apparent high rates of lifelong use of psychiatric care, 22.9% of people with a PMAD diagnosis had never sought care. The percentages of non-use did not differ among diagnoses: 25.6% for psychotic, 28% for severe mood, 22.2% for anxiety and 18.2% for mild mood disorders.

On multivariate logistic regression analysis, lifetime healthcare use was significantly associated with increased educational level (OR = 4.7, 95% CI: 1.4−15.3, *p* = 0.01), suicidal risk at the time of the survey (OR = 3.9; 95% CI: 1.1−13.4, *p* = 0.03) and a personality disorder (OR = 4.9, 95% CI: 1.1−22.2, *p* = 0.04) (Table 2). Lifetime healthcare use was reduced with living in a hotel vs. an emergency center at the time of the study (OR = 0.4, 95% CI: 0.1−1.0, *p* = 0.006).

### 3.2. Current Follow-Up

Only 18.2% of homeless people with a PMAD (*n* = 377, N = 9762) were being followed up at the time of the survey (Table 1), with a significant difference by diagnosis; taking into account comorbidities, people with a psychotic disorder were more frequently followed up than others were (*p* < 0.05).

Homeless people being followed up consulted mainly in the public system: 37% in medico-psychological centers and 23% in hospitals but also significantly in private practice (25%). Less than 10% were followed up in association or accommodation centers. Among those followed up, 63.3% had taken a treatment in the last month, with no significant difference by diagnostic group.

On the multivariate logistic regression analysis, the factors associated with currently being followed up (Table 3) were followed for a somatic chronic disease (OR = 3.2, 95% CI: 1.1−9.7, *p* = 0.04), social security coverage (OR = 5.7, 95% CI: 1.4−22.4, *p* = 0.01), perception of psychic suffering (OR = 3.7, 95% CI: 1.1−12.7, *p* = 0.03), and having a psychotic disorder (OR = 8.3, 95% CI: 1.5−44.7, *p* = 0.002). In contrast, an age ≥ 42 years was associated with reduced probably of being followed up (OR = 0.1, 95% CI: 0.0−0.5, *p* = 0.02).

### 3.3. Break in Care

Among the homeless individuals with a PMAD (Table 1) and a lifetime utilization of mental health services (*n* = 232, N = 6705), 71.8% were “out of care”—that is, not properly followed at the time of the survey. Individuals with a psychotic disorder were significantly less “out of care” than were others, although this rate remained high (41.8%) (*p* < 0.05).

### 3.4. Non-Compliance with Treatment

In our study, 40.4% of homeless people with a PMAD and a history of psychiatric medication (*n* = 153, N = 4390) had already stopped the prescribed treatment before the end of treatment (Table 1); individuals with a psychotic disorder stopped more frequently than others (*p* < 0.05). The reasons for this lack of compliance were first lack of need (26.8%), then a perceived ineffectiveness of treatment (24.1%), causes related to difficulties taking medicine (19.5%) or side effects (18.1%). Only three people mentioned reasons that could be described as “related to the street”: financial or theft problems and fear of being seen. Among these non-compliant people (*n* = 86; N = 2282), 40.9% declared having taken the medication again afterwards—for 95.9% of them because they “felt too bad” and for 3.3% due to a better understanding of the disorder.

## 4. Discussion

Homeless people with a psychotic, mood or anxiety disorder in the Greater Paris area had a fairly high lifetime use of psychiatric care; indeed, 68.7% had consulted, received treatment, or been hospitalized for a mental health reason (Table 1). However, 22.9% stated not having been in contact with the healthcare system, and only 18.0% were being followed up at the time of the survey. People with a PMAD were not able to anchor themselves in care: 72.3% discontinued care after a first contact and 40.4% showed poor adherence to treatment. Socio-demographic factors (age ≥ 42 years, less education) or social factors (no social security coverage, living in hotels) hindered follow-up or lifetime use of healthcare, and certain medical factors (psychotic disorder, personality trouble, risk of suicide, somatic chronic illness, declaring mental suffering) facilitated the use of healthcare (Table 3).

Our study has some limitations. The main one is that we were not able to include the most disadvantaged people, who do not come into contact with support structures for homeless people. Considering that these people are very marginalized, this could have led to an overestimation of the use of care. However, Kovess et al. [15,33] showed with a small sample that these people differed very little from the poorest people included in their study. Moreover, previous [15] or following [34] studies in the Greater Paris area have shown that almost all the homeless people who do not use housing services, but live in the streets or in the public space, frequent hot meal distribution points (at least once in the week before the interview). In other words, the inclusion of hot meal distribution points allowed us to capture the quasi-entire homeless population that does not use housing services. Second, we analyzed data for the subsample of people with psychiatric disorders. In some analyses, this may have resulted in a lack of power to highlight the results described in the literature (such as the role of addictions associated with access to care) [15,35] and not allow certain analyses. A final limitation is that the notion of use of whole-life care takes into account all contacts with the care system for psychiatric reasons, whatever the date in relation to the current disorders, without judging the adequacy between need and use. This use, if high, does not guarantee that people have been able to receive adequate care. That is why we also used three other indicators.

Our study shows that a significant proportion of people with a PMAD had access to care in their lifetime for a reason related to mental health (68.2%). This rate does not statistically differ from the proportion in the Kovess et al. study (59.3%) [33], so we cannot conclude that this use of care has changed in 15 years. Likewise, our results are close to those found in the literature for this type of population [36]. Additionally, the lifetime hospitalization rate we found (34%) does not differ from that for homeless people with the same disorders in the literature (30%) [37].

Thus, homeless people seem to have access to healthcare, as noted by a French study reporting that homeless people “attend medicine and psychiatry as much as [in] the general population” [38]. International studies had the same observation [39] and indicated an even greater use than in the general population [40], particularly for hospitalization [41,42], for those with psychiatric disorders or not. Additionally, in the general population with mood and anxiety disorders, the lifetime use of any professional for a mental health problem was 34% in France, compared to 49.4% to 72% in our sample (Table 1) [43].

The use of lifetime as measurement for mental health is problematic [44,45,46] and it is probably wrong to make the connection between preserved access to care and relatively high rates of lifetime use. Indeed, the latter rates do not mean that homeless people do not encounter obstacles in accessing care because of their situation. Homeless people may be able to seek care before being in their current situation and/or for another disorder than the one identified at the time of the survey. Thus, we should be careful in interpreting the use of lifetime care, an indicator that regardless, remains useful to compare studies.

A result, perhaps less questionable, complementary to the previous one, is the absence of healthcare utilization by some homeless people: 22.9% of people with an identified disorder had never been in contact with the healthcare system for this trouble (Table 1). Likewise, we found a low declared follow-up rate: only 18.2% of people with a PMAD declared that they were currently receiving care for mental health reasons at the time of the survey. Of these people, almost two-thirds had taken treatment in the previous month. The actual follow-up rate was increased for people with psychotic disorders (36.5%) and reduced for those with anxiety disorders (7.4%). People with a psychotic disorder were followed significantly more frequently (*p* < 0.05) than people with depressive or anxiety disorders, a result already described in this population [36] and in the general population [10]. This contradiction between high whole-life use rate, greater than in the general population, and low follow-up rate, was previously reported with the same proportions [21].

Concerning the factors associated with heath service use and follow-up, we found, as in the literature, that young people with a personality disorder [35], somatic chronic illness and suicide risk [15] seek more care. Social coverage also improves health service utilization [47]. Several studies, in the general population and among homeless people, have shown the role of sex in health service use, with women seeking more care than men [15,35]. We did not find this result perhaps because we took into account psychological suffering in analyses, which can be interpreted as a recognition of disorders. With the perception of symptoms being an important mediator of seeking care [36,48,49], would women seek more care because they perceive their pathology better or are they more naturally inclined to seek help [50]? Likewise, we did not find a role for social supports in the use of psychiatric care, contrary to other studies [51].

Our results on treatment adherence match those published in this type of population [47]. They are close to those observed in the general population [52] in terms of frequency and reasons for stopping the treatment. The reasons given in our study were not linked to the context of “life on the street”, even if this context should lead to changes in the prescriptions to improve compliance [53].

In addition to an analysis of access, we must determine whether homeless people, particularly those with somatic chronic disease, were able to enroll in the continuity of care. In our study, homeless individuals with mental health problems were unable to continue their healthcare because 72.3% had stopped the initialized care. Having a psychotic disorder reduced the risk of interruption of care; only 41.8% of people with a detected psychotic disorder interrupted their care. This medical “wandering” was already described in a Canadian study showing stable annual referral rates over the 5-year longitudinal survey, but the same people were not seen [54]. Subsequent studies are needed to study maintenance in care, specifically in people with psychiatric chronic disease (for which this notion takes on its full meaning) and to identify the disruptive factors (ignorance of the disorder, social difficulties, etc.).

Should our results be interpreted as a failure to care? To try to answer this question, we must first clarify the notion of need for care. Indeed, to speak of a lack of care is to implicitly say that care should have been given, that there is an unmet need for care. However, in general, epidemiological surveys only highlight “theoretical” needs (when they base the need for care on the sole diagnosis). Hence, several reasons can explain the lack of people seeking care (despite a diagnosis established). On the one hand, the tools used to detect symptoms or diseases in epidemiological investigations have “population” validity but do not, of course, allow for individual diagnoses, especially in psychiatry. On the other, the disorder thus highlighted is not necessarily in line with the need perceived and expressed by people themselves (or those around them); the expressed need is collected rather by questions on the renunciation of care [55]. However, in many psychiatric illnesses (especially psychotic illnesses), denial is a clinical component of the symptomatic picture, and in these situations, the need and even the possibility of care are assessed on a case-by-case basis (from therapeutic abstention to compulsory treatment). In addition, the expression of a need for care, a symptom or a suffering should not be considered only in its spontaneous dimension; it can emerge from an interaction between the individual and a physician (yet, as we have seen, consulting a physician is not exceptional—far from it—in this population). Then, an absence of health service utilization (and follow-up) with a lack of perception of need cannot completely exonerate the care system.

Finally, the need for psychiatric care remains a fairly complex notion. Not everyone with a mental health problem needs care [56]. This need is assessed individually and on a case-by-case basis, particularly depending on the impact of the disease on the social roles and the daily life of the person or even their psychological distress [57]. Measuring a disorder does not necessarily imply a need for treatment [58]. Mild problems may not require treatment and resolve on their own. Conversely, a need for care can be based on criteria other than diagnostic criteria for the disease. The need for care can be viewed as a continuum, rather than healthy subjects not requiring care and pathological subjects requiring care [59]. Healthcare access involves the recognition of the disorders and a request for care resulting from an assessment of the severity, the impact of the disorders, the acceptability of the treatment, the diagnosis and the existence of an effective treatment according to a benefit/risk balance.

The presence of disorders alone is not sufficient to define a need for care. Disabling symptoms and psychological distress must also be associated with it [60]. The factors that influence the use of care depend on the disease itself, its severity, its duration, a particular symptomatic profile, and the degree of impact on everyday life.

All these precautions are important to remember to avoid an overestimation of the non-response to needs based solely on epidemiological data. Indeed, the frequency of cases identified in an epidemiological survey and the rate of care utilization notably differ. This situation is quite common in mental health surveys, especially when they use standardized interview tools without a clinician [61]. For example, in a general population, we found rates of use for depression ranging from 30% to 65% depending on the severity of the episode in France [7,8,9,62,63] and internationally [64,65,66]. More generally, in an Australian study, only 35% of people with identified psychiatric disorders had consulted within 12 months [60] and in another study one- to two-thirds of psychiatric cases deemed severe were untreated [2]. So as Thornicroft wrote [67], most people with a mental disorder (67–74% depending on the study) do not receive treatment.

Our survey design theoretically minimized the frequency of false cases (we supplemented the standardized tool with the diagnosis by two clinicians), but the proportion of cases requiring treatment unfortunately remains unknown. Further studies are needed to better understand what, in the absence of heath service utilization, amounts on the one hand to a lack of perception of a real need (but, once again, this lack of perception does not completely implicate the practitioner or the healthcare system, whose role is also to detect and raise awareness of certain symptoms) and on the other to mental health problems that do not require care, and finally to genuine difficulties in access to care and follow-up, linked to the living conditions and lived experiences of homeless people [68].

Despite this, the actual follow-up rates observed in SAMENTA for people with a PMAD can only alert us: their weakness (including for severe disorders) is such that they cannot be explained only by the limits that we have just exposed.

## 5. Conclusions

Homeless people with psychiatric disorders have a non-negligible rate of lifetime use of care, even higher than the general population, especially in terms of hospitalization. As compared with the follow-up rate, if these people had access to the healthcare system at one point, they were unable to maintain care, which reflects a certain medical wandering. These difficulties, inherent in psychiatry, are certainly aggravated by homelessness.

How to improve the situation? The factors of unmet care and lack of follow-up are socio-demographic, socio-economic and psychiatric. A health action to guarantee the use of care can be addressed with the last two factors. The organization of care could be improved to reach vulnerable people who do not have access to care, those older than 40 years, and those living in hotels. The articulation between the various stakeholders could be improved, which would allow for continued care and avoid too-frequent emergency department visits [69]. We could focus our efforts on patient education to allow for continuity of care in a physician–patient therapeutic alliance and in respect of consent to care. We could develop the process of determining patient care needs so that users of services based on self-perceived care would have better outcomes [70,71]. Finally, we could provide housing to these people, the first factor of stability [72], which will allow them to develop their social rehabilitation [73].

## Figures and Tables

**Table 1:** Use of care for mental health by homeless people with a mental health diagnosis by diagnosis, Greater Paris Area (France), 2009.

	Total (%) (*n* = 377; N = 9762)	Psychotic Disorder (%) (*n* = 88; N = 2798)	Severe Mood Disorder (%) (*n* = 67; N = 1414)	Anxiety Disorder (%) (*n* = 112; N = 2535)	Non-Severe Mood Disorder (%) (*n* = 110; N = 3014)	*p* Value Comparing Diagnostic Groups
**Lifetime psychiatric use of care**	68.7	68.3	70.4	72.4	65.1	NS
Medical consultation	63.6	68.2	70.7	72.1	49.4	NS
Taking medication	40.1	47.7	50.9	28.8	37.4	NS
Hospitalization in a psychiatric unit	25.2	36.9	16.1	4.7	36.0	<0.05
Compulsory hospitalization	17.7	34.1	4.1	2.9	21.2	<0.05
**Current follow-up**	18.2	36.5	12.5	7.4	12.4	<0.05
Medication in the last month ^a^	63.0	75.2	78.2	67.4	20.9	NS
**Break in care ^b^**	72.3	41.8	82.2	89.8	80.6	NS
**Non-compliance with treatment ^c^**	40.4	61.3	23.6	17.2	38.9	<0.05

Results are weighted percentage, *n*—unweighted number of people studied, N—weighted number estimated in the population and NS—non-significant result; ^a^ percentages are calculated for the 86 people followed up (N = 1760): 37 with a psychotic disorder (N = 1022), 18 with a severe mood disorder (N = 177), 18 with an anxiety disorder (N = 177) and 13 with a mild mood disorder (N = 3014); ^b^ percentages are calculated for the 232 people with a lifetime psychiatric use of care (N = 6705), 60 with a psychotic disorder (N = 1912), 43 with a severe mood disorder (N = 996), 64 with an anxiety disorder (N = 1837) and 65 with a non-severe mood disorder (N = N 1961); ^c^ percentages are calculated for the 153 (N = 4390) people who had a medical prescription for treatment, 47 with a psychotic disorder (N = 1579), 30 with a severe mood disorder (N = 847), 30 with an anxiety disorder (N = 423) and 45 with a non-severe mood disorder (N = N 1541).

**Table 2:** Predictors of lifetime use of psychiatric care for homeless respondents with a mental health diagnosis (*n* = 377; N = 9762), Greater Paris area (France), 2009.

	OR	95% CI	*p* Value
Age, years			0.57
18−30	Ref.		
31−41	0.5	0.2–1.7	
42−82	0.7	0.2–2.7	
Female sex	0.5	0.2–1.9	0.34
Education level greater than secondary	4.7	1.4–15.3	0.01
Meeting place			0.006
Emergency center	Ref.		
Hotel	0.4	0.1–1.0	
Reintegration center	2.1	0.7–6.2	
Suicidal risk	3.9	1.1–13.4	0.03
Personality disorder	4.9	1.1−22.2	0.04
Diagnosis			0.30
Non-severe mood disorder	Ref.		
Psychotic disorder	0.8	0.3–2.6	
Severe mood disorder	0.5	0.1–2.2	
Anxiety disorder	1.6	0.7–3.7	

OR, odds ratio; 95% CI, 95% confidence interval.

**Table 3:** Predictors of psychiatric follow-up for homeless respondents with a mental health diagnosis (*n* = 377; N = 9762), Greater Paris area (France), 2009.

	OR	95% CI	*p* Value
Age, years			0.02
18−30	Ref.		
31−41	0.4	0.2–1.3	
42−82	0.1	0.0–0.5	
Female sex	2.5	0.7–8.3	0.14
Education level greater than secondary	2.6	0.4–15.9	0.30
Social security coverage	5.7	1.4–22.4	0.01
Meeting place			0.13
Emergency center	Ref.		
Hotel	0.2	0.0–0.9	
Reintegration center	0.8	0.2–2.7	
Feeling lonely	2.0	0.7–6.0	0.19
Follow-up for somatic chronic disease	3.2	1.1−9.7	0.04
Perception of psychic suffering	3.7	1.1−12.7	0.03
Diagnosis			0.002
Non-severe mood disorder	Ref.		
Psychotic disorder	8.3	1.5–44.7	
Severe mood disorder	0.8	0.1–4.5	
Anxiety disorder	0.9	0.7–3.7	

OR, odds ratio; 95% CI, 95% confidence interval.

## References

[1] Demyttenaere K., Bruffaerts R., Posada-Villa J., Gasquet I., Kovess V., Lepine J.P., Angermeyer M.C., Bernert S., de Girolamo G., Morosini P. (2004). Prevalence, severity, and unmet need for treatment of mental disorders in the World Health Organization World Mental Health Surveys. JAMA.

[2] Bijl R.V., de Graaf R., Hiripi E., Kessler R.C., Kohn R., Offord D.R., Ustun T.B., Vicente B., Vollebergh W.A., Walters E.E. (2003). The prevalence of treated and untreated mental disorders in five countries. Health Aff..

[3] Alegria M., Bijl R.V., Lin E., Walters E.E., Kessler R.C. (2000). Income differences in persons seeking outpatient treatment for mental disorders: A comparison of the United States with Ontario and The Netherlands. Arch. Gen. Psychiatry.

[4] Wells J.E., Robins L.N., Bushnell J.A., Jarosz D., Oakley-Browne M.A. (1994). Perceived barriers to care in St. Louis (USA) and Christchurch (NZ): Reasons for not seeking professional help for psychological distress. Soc. Psychiatry Psychiatr. Epidemiol..

[5] Jorm A.F., Christensen H., Medway J., Korten A.E., Jacomb P.A., Rodgers B. (2000). Public belief systems about the helpfulness of interventions for depression: Associations with history of depression and professional help-seeking. Soc. Psychiatry Psychiatr. Epidemiol..

[6] Jorm A.F., Korten A.E., Jacomb P.A., Rodgers B., Pollitt P., Christensen H., Henderson S. (1997). Helpfulness of interventions for mental disorders: Beliefs of health professionals compared with the general public. Br. J. Psychiatry.

[7] Kovess-Masfety V., Alonso J., Brugha T.S., Angermeyer M.C., Haro J.M., Sevilla-Dedieu C. (2007). Differences in lifetime use of services for mental health problems in six European countries. Psychiatr. Serv..

[8] Chan Chee C., Beck F., Sapinho D. (2009). La dépression en France-Enquête Anadep 2005.

[9] Wang P.S., Aguilar-Gaxiola S., Alonso J., Angermeyer M.C., Borges G., Bromet E.J., Bruffaerts R., de Girolamo G., de Graaf R., Gureje O. (2007). Use of mental health services for anxiety, mood, and substance disorders in 17 countries in the WHO world mental health surveys. Lancet.

[10] Shapiro S., Skinner E.A., Kessler L.G., Von Korff M., German P.S., Tischler G.L., Leaf P.J., Benham L., Cottler L., Regier D.A. (1984). Utilization of health and mental health services. Three Epidemiologic Catchment Area sites. Arch. Gen. Psychiatry.

[11] Stenius-Ayoade A., Haaramo P., Erkkila E., Marola N., Nousiainen K., Wahlbeck K., Eriksson J.G. (2017). Mental disorders and the use of primary health care services among homeless shelter users in the Helsinki metropolitan area, Finland. BMC Health Serv. Res..

[12] Codony M., Alonso J., Almansa J., Bernert S., de Girolamo G., de Graaf R., Haro J.M., Kovess V., Vilagut G., Kessler R.C. (2009). Perceived need for mental health care and service use among adults in Western Europe: Results of the ESEMeD project. Psychiatr. Serv..

[13] Susser E., Conover S., Struening E.L. (1990). Mental Illness in the homeless: Problems of epidémiologic method in surveys of the 1980s. Community Ment. Health J..

[14] Smith C., Castaneda E. (2020). Sick Enough? Mental Illness and Service Eligibility for Homeless Individuals at the Border. Soc. Sci..

[15] Kovess V., Mangin Lazarus C. (1999). The prevalence of psychiatric disorders and use of care by homeless people in Paris. Soc. Psychiatry Psychiatr. Epidemiol..

[16] Fazel S., Khosla V., Doll H., Geddes J. (2008). The prevalence of mental disorders among the homeless in western countries: Systematic review and meta-regression analysis. PLoS Med..

[17] Opalach C., Romaszko J., Jaracz M., Kuchta R., Borkowska A., Bucinski A. (2016). Coping Styles and Alcohol Dependence among Homeless People. PLoS ONE.

[18] Romaszko J., Kuchta R., Opalach C., Bertrand-Bucinska A., Romaszko A.M., Giergielewicz-Januszko B., Bucinski A. (2017). Socioeconomic Characteristics, Health Risk Factors and Alcohol Consumption among the Homeless in North-Eastern Part of Poland. Cent. Eur J. Public Health.

[19] Gelberg L., Gallagher T.C., Andersen R.M., Koegel P. (1997). Competing priorities as a barrier to medical care among homeless adults in Los Angeles. Am. J. Public Health.

[20] Lasser K.E., Himmelstein D.U., Woolhandler S. (2006). Access to care, health status, and health disparities in the United States and Canada: Results of a cross-national population-based survey. Am. J. Public Health.

[21] North C.S., Smith E.M. (1993). A systematic study of mental health services utilization by homeless men and women. Soc. Psychiatry Psychiatr. Epidemiol..

[22] Wen C.K., Hudak P.L., Hwang S.W. (2007). Homeless people’s perceptions of welcomeness and unwelcomeness in healthcare encounters. J. Gen. Intern. Med..

[23] Lewis J.H., Andersen R.M., Gelberg L. (2003). Health care for homeless women. J. Gen. Intern. Med..

[24] Hwang S.W., Ueng J.J., Chiu S., Kiss A., Tolomiczenko G., Cowan L., Levinson W., Redelmeier D.A. (2010). Universal health insurance and health care access for homeless persons. Am. J. Public Health.

[25] Parizot I., Chauvin P. (2003). The access to care of underserved populations: A research among free clinics patients in the Paris area. Rev. Epidemiol Sante Publique.

[26] Gallagher T.C., Andersen R.M., Koegel P., Gelberg L. (1997). Determinants of regular source of care among homeless adults in Los Angeles. Med. Care.

[27] Langle G., Egerter B., Albrecht F., Petrasch M., Buchkremer G. (2005). Prevalence of mental illness among homeless men in the community—Approach to a full census in a southern German university town. Soc. Psychiatry Psychiatr. Epidemiol..

[28] Baggett T.P., O’Connell J.J., Singer D.E., Rigotti N.A. (2010). The unmet health care needs of homeless adults: A national study. Am. J. Public Health.

[29] ARS (2012). Projet Régional de Santé d’Ile de France.

[30] Laporte A., Douay C., Detrez M.-A., Le Masson V., Le Méner E., Chauvin P. (2010). La Santé Mentale et les Addictions chez les Personnes Sans Logement Personnel d’Île-de-France.

[31] Laporte A., Vandentorren S., Detrez M.A., Douay C., Le Strat Y., Le Mener E., Chauvin P., Samenta Research G. (2018). Prevalence of Mental Disorders and Addictions among Homeless People in the Greater Paris Area, France. Int. J. Environ. Res. Public Health.

[32] Ardilly P., Le Blanc D. (2001). Sampling and weighting a survey of homeless persons: A French example. Surv. Methodol..

[33] Kovess V., Mangin Lazarus C. (2001). Use of Mental Health Services by Homeless People in Paris. Int. J. Mental Health.

[34] Arnaud A., Chosidow O., Detrez M.A., Bitar D., Huber F., Foulet F., Le Strat Y., Vandentorren S. (2016). Prevalences of scabies and pediculosis corporis among homeless people in the Paris region: Results from two randomized cross-sectional surveys (HYTPEAC study). Br. J. Dermatol.

[35] Bonin J.P., Fournier L., Blais R. (2007). Predictors of mental health service utilization by people using resources for homeless people in Canada. Psychiatr. Serv..

[36] Koegel P., Sullivan G., Burnam A., Morton S.C., Wenzel S. (1999). Utilization of mental health and substance abuse services among homeless adults in Los Angeles. Med. Care.

[37] Ducq H., Guesdon I., Roelandt J.L. (1997). Mental health of homeless persons. Critical review of the Anglo-Saxon literature. Encephale.

[38] Guesdon I., Roelandt J.L. (1998). Enquête lilloise sur la santé mentale des personnes sans domicile fixe. L’information Psychiatr..

[39] Herrman H., Evert H., Harvey C., Gureje O., Pinzone T., Gordon I. (2004). Disability and service use among homeless people living with psychotic disorders. Aust. N. Z. J. Psychiatry.

[40] Robins L.N., Locke B.Z., Regier D.A. (1991). Psychiatric Disorders in America: The Epidemiologic Catchment Area Study. An Overview of Psychiatric Disorders in America.

[41] Folsom D.P., Hawthorne W., Lindamer L., Gilmer T., Bailey A., Golshan S., Garcia P., Unutzer J., Hough R., Jeste D.V. (2005). Prevalence and risk factors for homelessness and utilization of mental health services among 10,340 patients with serious mental illness in a large public mental health system. Am. J. Psychiatry.

[42] Forchuk C., Brown S.A., Schofield R., Jensen E. (2008). Perceptions of health and health service utilization among homeless and housed psychiatric consumer/survivors. J. Psychiatr. Ment. Health Nurs..

[43] Dezetter A., Briffault X., Alonso J., Angermeyer M.C., Bruffaerts R., de Girolamo G., De Graaf R., Haro J.M., Konig H.H., Kovess-Masfety V. (2011). Factors associated with use of psychiatrists and nonpsychiatrist providers by ESEMeD respondents in six European countries. Psychiatr. Serv..

[44] Anderson L., Snow D.A. (2001). Inequality and the self: Exploring connections frome an interactionist perspective. Symb. Interact..

[45] Snow D., Anderson L., Koegel P. (1994). Distorting tendancies in research on the homeless. Am. Behav. Sci..

[46] Snow D., Baker S., Anderson L., Martin M. (1986). The myth of pervasive mental illness among the homeless. Soc. Probl..

[47] Kushel M.B., Vittinghoff E., Haas J.S. (2001). Factors associated with the health care utilization of homeless persons. JAMA.

[48] Koopmans G.T., Lamers L.M. (2007). Gender and health care utilization: The role of mental distress and help-seeking propensity. Soc. Sci. Med..

[49] Herman D., Elmer S., Barrow S. (1993). Self-assessed need for mental health services among homeless adults. Hosp. Community Psychiatry.

[50] Mackenzie C.S., Gekoski W.L., Knox V.J. (2006). Age, gender, and the underutilization of mental health services: The influence of help-seeking attitudes. Aging Ment. Health.

[51] Lam J.A., Rosenheck R. (1999). Social support and service use among homeless persons with serious mental illness. Int J. Soc. Psychiatry.

[52] Corruble E., Hardy P. (2003). L’observance du traitement en psychiatrie. Encyclopédie Médico-Chirurgicale, Pyschiatrie.

[53] Mercuel A. (2008). Les antipsychotiques auprès des usagers: Images et vecu en précarité. L’encephale.

[54] Bonin J.P., Fournier L., Blais R., Perreault M., White N.D. (2010). Health and mental health care utilization by clients of resources for homeless persons in quebec city and montreal, Canada: A 5-year follow-up study. J. Behav Health Serv. Res..

[55] Bazin F., Parizot I., Chauvin P. (2005). Original approach to the individual characteristics associated with forgone healthcare: A study in underprivileged areas, Paris region, France, 2001–2003. Eur. J. Public Health.

[56] Kovess V., Rouillon J.-D.G.e.F. (2007). Evaluation du besoin de soin en psychiatrie. Manuel de psychiatrie.

[57] Lovell A. (2003). Etude sur la surveillance dans le champ de la santé mentale.

[58] Spitzer R.L. (1998). Diagnosis and need for treatment are not the same. Arch. Gen. Psychiatry.

[59] Lin E., Goering P.N., Lesage A., Streiner D.L. (1997). Epidemiologic assessment of overmet need in mental health care. Soc. Psychiatry Psychiatr. Epidemiol..

[60] Andrews G., Henderson S., Hall W. (2001). Prevalence, comorbidity, disability and service utilisation. Overview of the Australian National Mental Health Survey. Br. J. Psychiatry.

[61] Le Pape A., Lecompte T. (1999). Prévalence et prise en charge médicale de la dépression en 1996–1997. Quest. d’economie St..

[62] Embersin C., Grémy I. (2008). La dépression chez les adultes franciliens. Exploitation du Baromètre santé 2005. 2008. ORS Ile de France. https://www.ors-idf.org/nos-travaux/publications/la-depression-chez-les-adultes-franciliens.html.

[63] Morvan Y., Prieto A., Briffault X., Blanchet A., Dardennes R., Rouillon F., Beck F., Guilbert P., Gautier A. (2007). La dépression: Prévalence, facteurs associés et consommation de soins. Baromètre santé 2005.

[64] Hamalainen J., Isometsa E., Laukkala T., Kaprio J., Poikolainen K., Heikkinen M., Lindeman S., Aro H. (2004). Use of health services for major depressive episode in Finland. J. Affect. Disord.

[65] Bijl R.V., Ravelli A. (2000). Psychiatric morbidity, service use, and need for care in the general population: Results of The Netherlands Mental Health Survey and Incidence Study. Am. J. Public Health.

[66] Sareen J., Cox B.J., Afifi T.O., Yu B.N., Stein M.B. (2005). Mental health service use in a nationally representative Canadian survey. Can. J. Psychiatry.

[67] Thornicroft G. (2007). Most people with mental illness are not treated. Lancet.

[68] Krausz R.M., Clarkson A.F., Strehlau V., Torchalla I., Li K., Schuetz C.G. (2013). Mental disorder, service use, and barriers to care among 500 homeless people in 3 different urban settings. Soc. Psychiatry Psychiatr. Epidemiol..

[69] Henry J.M., Boyer L., Belzeaux R., Baumstarck-Barrau K., Samuelian J.C. (2010). Mental disorders among homeless people admitted to a French psychiatric emergency service. Psychiatr. Serv..

[70] Lovell A.M. (1993). Assessment of needs and evaluation of interventions in mental health. Current approach. Rev. Epidemiol. Sante Publique.

[71] Bonabi H., Muller M., Ajdacic-Gross V., Eisele J., Rodgers S., Seifritz E., Rossler W., Rusch N. (2016). Mental Health Literacy, Attitudes to Help Seeking, and Perceived Need as Predictors of Mental Health Service Use: A Longitudinal Study. J. Nerv. Ment Dis..

[72] Girard V., Stecahandy P., Chauvin P. (2010). La Santé des Personnes Sans Chez Soi: Plaidoyer et Propositions Pour un Accompagnement des Personnes à un Rétablissement Social et Citoyen.

[73] Aubry T., Bourque J., Goering P., Crouse S., Veldhuizen S., LeBlanc S., Cherner R., Bourque P.E., Pakzad S., Bradshaw C. (2019). A randomized controlled trial of the effectiveness of Housing First in a small Canadian City. BMC Public Health.

